# Population genetics of ectoparasitic mites *Varroa* spp. in Eastern and Western honey bees

**DOI:** 10.1017/S003118201900091X

**Published:** 2019-07-31

**Authors:** Vincent Dietemann, Alexis Beaurepaire, Paul Page, Orlando Yañez, Ninat Buawangpong, Panuwan Chantawannakul, Peter Neumann

**Affiliations:** 1 Agroscope, Swiss Bee Research Center, Bern, Switzerland; 2 Department of Ecology and Evolution, University of Lausanne, Lausanne, Switzerland; 3 INRA, UR 406 Abeilles et Environnement, Avignon, France; 4 Molecular Ecology Group, Martin-Luther Universität Halle-Wittenberg, Halle/Saale, Germany; 5 Institute of Bee Health, Vetsuisse Faculty, University of Bern, Bern, Switzerland; 6 Bee protection laboratory, Department of Biology, Faculty of Science, Chiang Mai University, Chiang Mai, Thailand; 7 Environmental Science Research Center, Faculty of Science, Chiang Mai University, Chiang Mai 50200, Thailand

**Keywords:** *Apis cerana*, *Apis mellifera*, coevolution, host specificity, host–parasite interactions, hybridization, introgression, population genetics, *Varroa destructor*, *Varroa jacobsonii*

## Abstract

Host shifts of parasites are often causing devastating effects in the new hosts. The *Varroa* genus is known for a lineage of *Varroa destructor* that shifted to the Western honey bee, *Apis mellifera*, with disastrous effects on wild populations and the beekeeping industry. Despite this, the biology of *Varroa* spp. remains poorly understood in its native distribution range, where it naturally parasitizes the Eastern honey bee, *Apis cerana*. Here, we combined mitochondrial and nuclear DNA analyses with the assessment of mite reproduction to determine the population structure and host specificity of *V. destructor* and *Varroa jacobsonii* in Thailand, where both hosts and several *Varroa* species and haplotypes are sympatric. Our data confirm previously described mite haplogroups, and show three novel haplotypes. Multiple infestations of single host colonies by both mite species and introgression of alleles between *V. destructor* and *V. jacobsonii* suggest that hybridization occurs between the two species. Our results indicate that host specificity and population genetic structure in the genus *Varroa* is more labile than previously thought. The ability of the host shifted *V. destructor* haplotype to spillback to *A. cerana* and to hybridize with *V. jacobsonii* could threaten honey bee populations of Asia and beyond.

## Introduction

Host shifts of parasites can lead to biological invasions and result in emerging infectious diseases with devastating effects on the populations of the new hosts (Pimentel *et al*., 2005; Kumschick *et al*., 2015; Wells and Clark, 2019). A better knowledge of the drivers of host shifts in the natural distribution areas of parasites could help mitigating their negative effects, preventing future invasions (Kolar and Lodge, 2001; Woolhouse *et al*., 2005) and can contribute to a better understanding of the coevolution between hosts and parasites (Thompson, 1994). Host shifts can be promoted by high parasite genetic diversity, low host specificity and by introgression between species (Longdon *et al*., 2014; Depotter *et al*., 2016; Wells and Clark, 2019), all of which can be studied using molecular tools (Criscione *et al*., 2005; de Meeus *et al*., 2007).

The Western honey bee, *Apis mellifera*, is a good model species to study host shifts. Because of the pollination service it provides and its economic importance (Klein *et al*., 2007; Kleijn *et al*., 2015), colonies of this social insect have been translocated to where beekeepers deemed appropriate and beneficial. Consequently, *A. mellifera* has been introduced to ecosystems beyond its natural distribution range and frequently exposed to parasites and pathogens never encountered before. In Asia, the ectoparasitic mite *Varroa destructor* successfully shifted to *A. mellifera* following its introduction into territories occupied by the Eastern honey bee, *Apis cerana*, the original host of this parasite (Rosenkranz *et al*., 2010). Lacking the necessary adaptive mechanisms against the parasite, most *A. mellifera* populations are unable to survive infestations, with negative consequences climaxing in colony failure within a few years (Rosenkranz *et al*., 2010). Subsequently, *V. destructor* has become the most detrimental biotic threat to *A. mellifera* by negatively affecting the development of honey bee brood, on which the parasite feeds and reproduces (Rosenkranz *et al*., 2010), and by transmitting viruses (Wilfert *et al*., 2016). This pest has led to the near eradication of wild *A. mellifera* populations in the Northern hemisphere (Le Conte *et al*., 2007; Jaffé *et al*., 2009) and to high losses of managed colonies worldwide (Genersch *et al*., 2010; Guzmán-Novoa *et al*., 2010; Le Conte *et al*., 2010; Nguyen *et al*., 2011; Smith *et al*., 2013) with high economical and societal costs (Kumschick *et al*., 2015).

Since *V. destructor* invaded Europe and the Americas in the 1970s and 1980s, an intense research activity on its biology in *A. mellifera* has been undertaken with the main aim of finding effective control methods to protect colonies (Rosenkranz *et al*., 2010; Dietemann *et al*., 2012). Comparatively, little attention has been devoted to the interaction between *Varroa* spp. mites and their original host, *A. cerana* (Dietemann *et al*., 2012; Wang *et al*., 2018), despite the fact that several other mite haplotypes shifted host (Beaurepaire *et al*., 2015; Roberts *et al*., 2015). Although they did not yet lead to new large-scale invasions, these new shifts show the propensity of the mite genus to generate more ecological and economic problems. Even though high genetic diversity has been shown in the genus *Varroa* (Anderson and Fuchs, 1998; de Guzman *et al*., 1998; Anderson and Trueman, 2000; Warrit *et al*., 2006; Navajas *et al*., 2010; Beaurepaire *et al*., 2015; Roberts *et al*., 2015), little knowledge currently exists on host specificity and their potential to hybridize, making it difficult to evaluate risks for new host shifts and invasions. Indeed, previous studies in the endemic range of *Varroa* spp. rarely reported whether the mites collected were reproducing in their host brood, preventing a systematic evaluation of host specificity (see Roberts *et al*., 2015 for an exception). In addition, the genetic markers used to define species and haplotype distribution of these mites (i.e. mitochondrial markers), do not allow for the detection of introgression. Indeed, mitochondrial DNA is maternally inherited and only reflects maternal gene flow (Harrison, 1989), giving only a partial picture of population structure. Even though paternal transmission can seem insignificant due to the reproductive system of the *Varroa* mites (mother mites produce one son and several daughters that mate together in the brood cells, Rosenkranz *et al*., 2010), recent studies showed that paternal gene flow is not negligible. In fact, reproduction can occur between inbred lineages when occupying the same cell (Beaurepaire *et al*., 2017*a*). Therefore, the use of nuclear DNA markers such as microsatellites can help completing the picture by unravelling finer levels of genetic structuring of populations than mitochondrial DNA (Beaurepaire *et al*., 2015; Roberts *et al*., 2015).

Here, we studied the population genetic structure of *V. destructor* and *Varroa jacobsonii* mites in Thailand using both mitochondrial DNA and microsatellite markers to unravel phenomena promoting host shifts. In this country, the sympatric occurrence of the two hosts and several mite species (Warrit *et al*., 2006) leads to opportunities for host shifts. Yet, in the former study, a single mite was sampled per colony and a small fragment of the COI gene (328 bp) was used to determine the prevalence of mite haplotypes and species. We conducted a more intense sampling at a local scale and observed the ability of these mites to reproduce on the host they were collected from by monitoring their reproductive status. This allowed us to increase chances of detecting phenomena that promote host shifts (e.g. drifting of mites, introgression) or host shifts *per se* (e.g. reproduction in a new host's brood). Surveying the distribution of mitochondrial haplotypes in the same regions as Warrit *et al*. (2006) more than a decade later, we also assess temporal changes in population structure. Our results show that genetic structure and host specificity in the genus *Varroa* is more labile than previously thought. We detected the occurrence of several phenomena promoting host shifts, which could represent a threat to the honey bee populations of Asia and beyond.

## Methods

### Populations, sampling

**Fig. 1.**
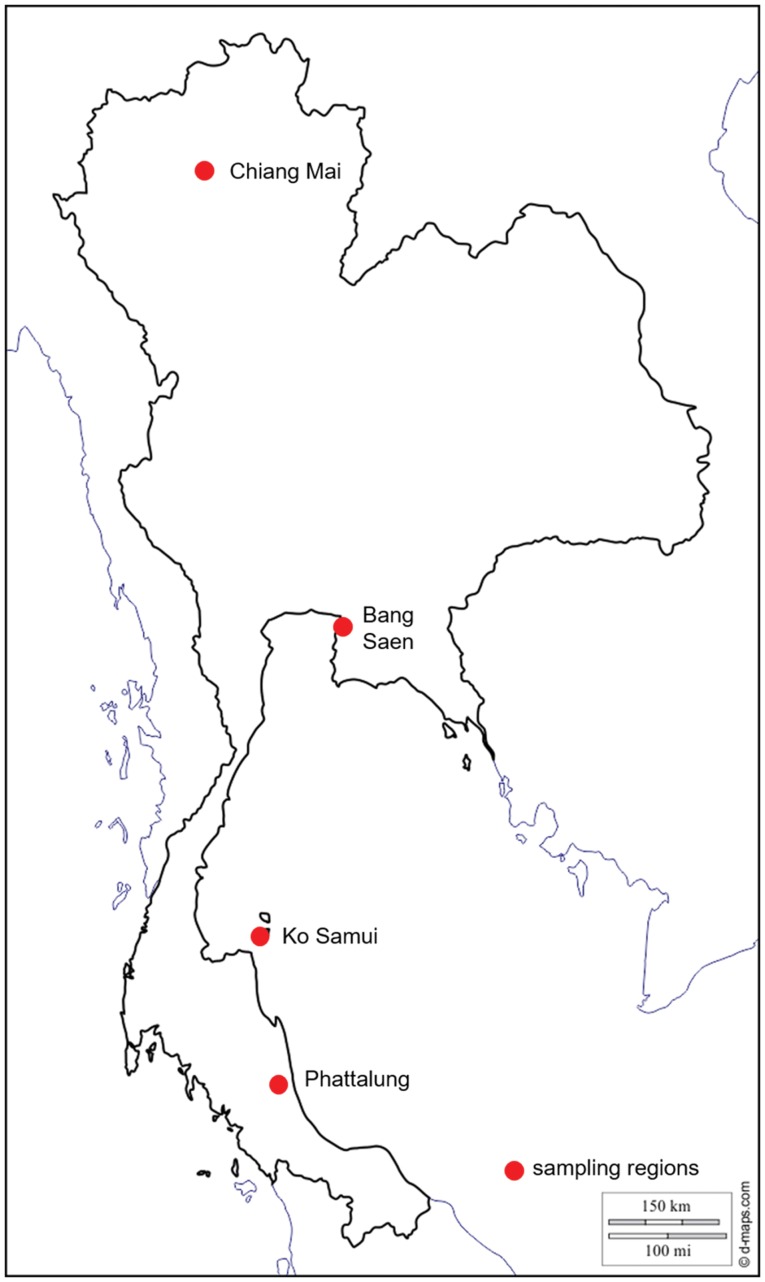
Map of Thailand showing the sampling locations (see Table 1). In the text, these locations are referred to as North for Chiang Mai, centre for Bang Saen, island for Ko Samui and South for Phattalung. *Apis mellifera* colonies were screened in the North and centre and *Apis cerana* at all locations.

Between 2013 and 2015, 200 *Varroa* spp. mites were collected from drone brood cells of *A. cerana* in one to five apiaries from four regions of Thailand (Table 1, Fig. 1): (1) Chiang Mai (North) where *V. jacobsonii* haplotype North Thai and *V. destructor* Vietnam were reported in *A. cerana* (above 1000 m for the latter); (2) Bang Saen (Chon Buri, central Thailand) with *V. jacobsonii* haplotype North Thai; (3) Ko Samui (island) with *V. jacobsonii* Samui; (4) Phattalung (South) with *V. jacobsonii* Malay (Warrit *et al*., 2006). North of the Isthmus of Kra (North and centre locations), *A. mellifera* hosting the host shifted lineage of *V. destructor* Korea can be found. A sample of 172 mites was thus collected from drone and worker brood cells of *A. mellifera* in Chiang Mai and Bang Saen (Table 1, Fig. 1). Although *A. mellifera* is occasionally kept south of the Isthmus, they do not survive there for long periods and have to be imported regularly from the North (P. Chantawannakul unpublished) and were therefore not screened in this region.

**Table 1.** Sampling region, host species of origin and number of *Varroa* spp. mites genotyped for mtDNA and microsatellites. The table also indicates the numbers of mite drifts between host species and of introgression events between mite species

Host species	Location (region)	mtDNA			Microsatellites					
		*N* mites (*N* colonies)	*N* drifts (*N* colonies)	*N* colonies infested by multiple haplotypes	*N* mites (*N* colonies)	*N* drifts (*N* colonies)	*N* colonies infested by multiple haplotypes	*N* likely + less likely hybrids (*N* colonies)	*N* likely hybrids (*N* colonies)	N hybrids identified by Visual inspection
*A. mellifera*	Chiang Mai (North)	117 (10)	1 (1)	0	26 (7)	1 (1)	1	0	0	1
	Bang Saen (centre)	2 (2)	0	0	27 (3)	0	0	2 (1)	2 (1)	2
*A. cerana*	Chiang Mai (North)	65 (10)	8* + 1 (4)	4	34 (3)	5 (1)	1	1 (1)	1 (1)	1
	Bang Saen (centre)	3 (2)	0	0	16 (4)	0	0	1 (1)	1(1)	1
	Ko Samui (island)	20 (9)	0	0	12 (1)	0		0	0	0
	Phattalung (South)	18 (5)	0	0	32 (2)	0	0	6 (2)	1 (1)	12

Drift and introgression were identified based on mitochondrial DNA and microsatellite data. Introgression was first detected based on the Instruct results and then verified by visual inspection of the mite genotypes (Table S8). ‘Likely’ hybrids were identified based on a probability over 5% of belonging to the other cluster revealed by Instruct. A less conservative threshold set at 2.5% probability identified further ‘less likely’ hybrids.

### Mite reproductive status

Upon opening of infested host brood cells, the reproductive state of mite foundresses was determined (Dietemann *et al*., 2013). The occurrence of at least one offspring of any sex confirmed that the foundresses were fertile, unless host developmental stage preceded oviposition. These cases, together with infertile foundresses were considered as non-reproductive. Percentage of reproductive foundresses is reported out of the total number of foundress mites found (fertile and non-reproductive). Mites were placed in 75% EtOH and frozen at −20 °C until DNA extraction.

### DNA extraction

DNA of individuals used for sequencing were extracted with phenol-chloroform (*N* = 193; Evans *et al*., 2013) and with TaKaRa lysis buffer for microorganisms (*N* = 32; Takara Bio Inc., Otsu, Japan). For the latter, the tubes were heated at 65 °C for 30 min and then at 100 °C for 10 min before adding 40 *µ*L double distilled H_2_0. The tubes were then vortexed and centrifuged. Ten microlitres of 2X GenStar PCR-ready mix (with Taq + loading dye), 7 *µ*L double distilled H_2_0, 1 *µ*L forward, 1 *µ*L reverse primers and 1 *µ*L of DNA extract was added to PCR tubes. Total DNA of mites collected in Bang Saen (*n* = 51) was isolated according to Beaurepaire *et al*. (2017*a*). DNA of individuals used for microsatellite analyses (*N* = 164) were extracted with Chelex: the ethanol used to preserve the mites was rinsed twice in double distilled H_2_O and each mite was placed individually in 100 *µ*L 5% Chelex solution in a 96 well plate and crushed with a pestle. Finally, 5 *µ*L proteinase *K* were added and the plate was placed in a thermocycler with standard Chelex cycling conditions (Walsh *et al*., 1991).

### PCR amplification and sequencing

PCR assays were performed to amplify regions of the cytochrome oxidase subunit I (cox1) gene of the mites sampled from the North, South and island locations (Evans *et al*., 2013; Table S1). The analyses were performed by using MyTaq™ kit (Bioline, London, UK) following the manufacturer's recommendations. Briefly, 2 *µ*L 10-fold-diluted of the extracted DNA, 5X reaction buffer, forward and reverse primers (final concentration of 0.4 *µ*m each) and Taq polymerase (0.63 units) were mixed in 25 *µ*L final reaction volume. Primers given in Table S1 were used to produce 380 and 800 bp amplicons.

The PCR cycling protocols are given in Table S2. The PCR products were analysed with a 2% two-dimensional agarose gel electrophoresis. The gels were stained GelRed (Biotium, Hayward, CA, USA) for visualization under UV light. The PCR products were purified using the NucleoSpin^®^ Gel and PCR Clean-up kit (Macherey-Nagel, Co., Düren, Germany) following the manufacturer's recommendations. Purified PCR products were commercially sequenced. Each PCR product was sequenced in both directions.

### Haplotype network analyses

**Fig. 2.**
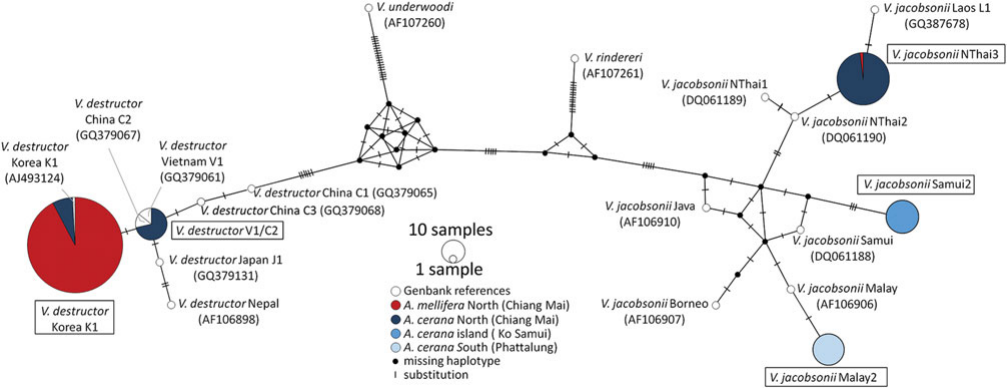
Haplotype Network (Median-Joining) based on mtDNA of *Varroa* spp. sampled in three regions of Thailand (Chiang Mai, Ko Samui, Phattalung, Table 1) and from reference collections (Anderson and Trueman, 2000; Warrit *et al*., 2006; Navajas *et al*., 2010). Haplotypes detected during the present survey are highlighted with a box. Reference samples are shown without boxes and followed by their accession numbers between parentheses. Host species of origin are coded with colours: red for *A. mellifera* and blue for *A. cerana*. Location latitude is coded with shades: dark to light from north to south.

A dataset including the overlapping region of the 380 and 800 bp sequences together with GenBank references (Anderson and Trueman, 2000; Warrit *et al*., 2006; Navajas *et al*., 2010) was generated (Fig. 2). Median-Joining haplotype networks of this 290 bp region were obtained in PopART with epsilon = 0 (http://popart.otago.ac.nz).

The sequence of one representative mite per host species and region was uploaded to GenBank with the accession numbers MN179648–MN179654.

### Microsatellite DNA analyses

*Varroa* spp. mites collected at all locations were genotyped at eight DNA microsatellite loci (VD305, VD307, VD112, VJ292, VJ294, Vdes-01, Vdes-02, Vdes-04; Evans, 2000; Solignac *et al*., 2005) using the Fragment Profiler software V. 1.2 of the MEGABACE DNA Analysis System (GE Healthcare Life Science, Buckinghamshire, England). Samples with missing information for more than three loci were excluded, resulting in a dataset of 147 mites (Table 1). Hardy–Weinberg equilibrium and linkage disequilibrium tests were performed within samples for each marker using Fstat V. 2.9.3 (Goudet, 1995).

### Identification of drifters

To identify putative drifters (mites found in colonies of an atypical host, i.e. *V. destructor* Korea in *A. cerana* and *V. jacobsonii* in *A. mellifera*), an analysis not relying on *a priori* information was conducted with the software InStruct (Gao *et al*., 2007). InStruct is an alternative to the software STRUCTURE (Pritchard *et al*., 2000) that can handle analyses of inbred or partially self-fertilizing species, as is the case for *Varroa* spp. The number of clusters in the dataset (*K*) varied from 1 to 12 and 20 chains for each run were performed with 50 000 burn-in iterations and 100 000 total iterations for each chain using the Admixture model. The most likely number of clusters was then estimated manually following the method described in Evanno *et al*. (2005). The results of the runs were combined with the software *CLUMPP* (Jakobsson and Rosenberg, 2007) and the software Distruct (Rosenberg, 2004) was used to draw the corresponding figures. To match the genotype clusters to known mite species using the nucleotide BLAST tool on the NCBI platform (Altschul *et al*., 1990), a subset of 15 mites used in the microsatellite analysis were sequenced with the 929 bp COI primer from Navajas *et al*. (2010). See section ‘Mitochondrial DNA analysis’ for methodology.

### Identification of hybrids

To identify hybrids between *V. destructor* and *V. jacobsonii*, the cluster membership probabilities of each individual over the 20 chains obtained for *K* = 2 with the software CLUMPP were compared. Individuals with a probability to belong to both clusters superior to 5% was considered as a likely hybrid. Any individual with a probability superior to 2.5% to belong to two clusters was considered as a less likely hybrid. Introgression of alleles was then confirmed based on the occurrence of heterospecific alleles, i.e. common to the two species. When the allele frequency in one of the species was 5-fold that of the same allele in the other species, we considered that the allele very likely belonged to the former species. When the ratio of allele frequencies was below five, we did not propose an origin for the allele.

### Analyses of the genetic diversity and population structure

To estimate the level of genetic diversity and population structure of the different *Varroa* taxa found in Thailand, all drifters identified by InStruct were removed from the dataset. The number of alleles (*N*_A_), allelic richness (*R*) and the expected (*H*_e_) and observed heterozygosity (*H*_o_) indexes were then calculated for each mite group sampling location using the software Fstat V. 2.9.3 (Goudet, 1995).

Several estimates of genetic differentiation (*F*_ST_, *G*_ST_, *D*_est_) were calculated using GenAlex V. 6.503 (Jost, 2008; Peakall and Smouse, 2012). *F*_ST_ index quantifies how the reduction in gene flow among populations affects their level of heterozygosity. In addition, it reflects the variance in allele frequencies for markers with two alleles. When markers have more than two alleles, interpreting *F*_ST_ is more challenging (Jost, 2008). We nevertheless report *F*_ST_ since it is the most commonly used estimate of the reduction in heterozygosity due to population structure in population genetics studies (Whitlock, 2011). To work around this bias, we also calculated *G*_ST_, which is a corrected estimate of *F*_ST_, adjusted for markers with more than two alleles (Nei, 1973). A third estimate, Jost's *D*_est_ (Jost, 2008) was calculated. It focuses on variance in allele frequencies among populations. Thus, we report the three estimates to provide complementary information on the genetic differences among the mites of the different populations as recommended in Meirmans and Hedrick (2011). In case several individuals sharing the same genotype were found in a colony, only one sample was included to estimate levels of genetic differentiation in order to avoid biasing the analysis with highly related individuals. This led to the exclusion of 25 individuals, leaving 116 for the analyses.

In addition, a pairwise distance-based AMOVA with 1000 permutations was performed for each species using the software Arlequin V.3.5.1.3 (Excoffier *et al*., 2005). These analyses were based on the microsatellite dataset without drifters but with putative hybrids and individuals sharing the same genotype to identify the distribution of genetic variation in each species.

Finally, a Principal Component Analysis (PCA) was conducted on the same dataset to identify the main genetic clusters among the mite samples. Since we were interested in the occurrence of putative hybrids, we performed the PCA including the data of both species. The R package Adegenet (Jombart, 2008) in R v. 3.5.2 (R core Team, 2018) was used.

## Results

### Mite distribution and reproduction

**Fig. 3.**
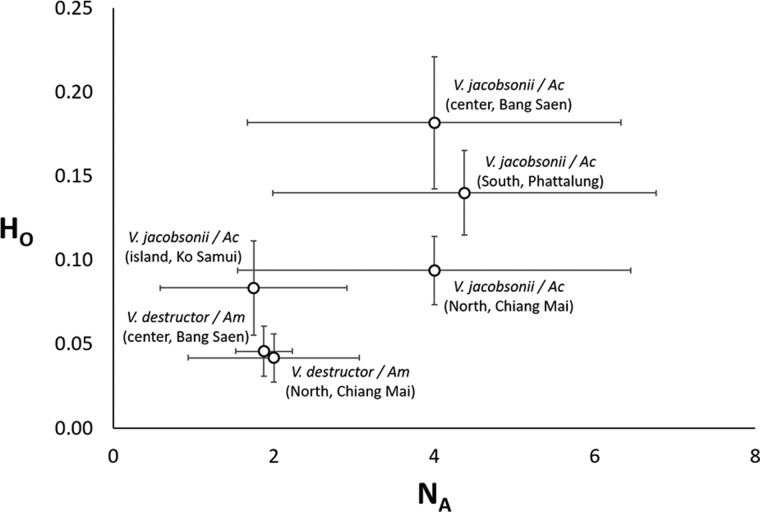
Average (±s.d.) allelic richness *vs* heterozygosity in *Varroa* spp. mite populations of two host species (Am: *Apis mellifera*, Ac: *Apis cerana*) in four regions of Thailand.

With a single exception, mitochondrial DNA sequences of the mites collected from *A. mellifera* colonies in North Thailand (*N* = 118) were identified as the *V. destructor* Korean haplotype 1 (K1) (Figs 2 and 3). Twenty eight of these mites (24%) had reproduced, while the remaining mites (*N* = 89) either had not reproduced or were collected from early host brood stages on which reproduction is not yet detectable. The exception was a non-reproductive *V. jacobsonii* mite in an *A. mellifera* colony (Table 1). This individual belonged to a novel haplotype that we named NorthThai3.

*Varroa destructor* K1 mites were also found in two years in ten drone brood cells of four *A. cerana* colonies in the North (Fig. 2). Eight of these mites (80%) had successfully produced offspring (Table 1). In *A. cerana* colonies screened in this region, we also found *V. destructor* of the Vietnam haplotype 1 (V1) or of the China haplotype 2 (C2, the region sequenced did not allow for discriminating between these haplotypes, Fig. 2). Three of the V1/C2 mites (60%) had reproduced in drone cells. The haplotype found most frequently (75%) in *A. cerana* colonies in the North was the newly described *V. jacobsonii* NorthThai3 (Fig. 2). It differed from NorthThai1 and NorthThai2 identified by Warrit *et al*. (2006) in two and one nucleotides, respectively, and from the Laos mite haplotype L1 in one nucleotide (Fig. 2). Twenty-six of these mites (53%) had reproduced on drone brood. In the South, the mites found in *A. cerana* colonies differed from the *V. jacobsonii* Malay haplotype (Warrit *et al*., 2006) by one nucleotide (Fig. 2). We named this new haplotype Malay2. Thirteen of these mites (72%) had produced offspring in infested drone cells. Similarly, the *V. jacobsonii* mite haplotype collected from *A. cerana* colonies on the island differed from that reported earlier. We found a difference of four nucleotides and this newly reported haplotype was called Samui2 (Fig. 2). Eleven of the Samui2 mites (55%) collected from drone brood were fertile. The haplotype network inferred several haplotypes that were not sampled during our screening.

MtDNA genotyping showed that four *A. cerana* colonies in the North were infested by the two mite species simultaneously (Table 1). One of these colonies was infested with *V. jacobsonii* NorthThai3 as well as with two haplotypes of *V. destructor* (K1 and V1/C2). Each of these mite species and haplotypes reproduced on drone brood in at least one occurrence in these four colonies.

### Genetic diversity and population structure of *Varroa* spp. in Thailand

Out of the eight microsatellites we used, none of the locus pairs was significantly linked after Bonferroni correction (Table S3). These markers were polymorphic, with a range of 3–16 alleles per locus (8.9 ± 4.3, mean ± s.d., Table S4). The average allelic richness per locus varied from 2.1 to 6.4 (4.7 ± 1.5, mean ± s.d., Table S5). With the exception of low values for *V. jacobsonii* Samui2, the two genetic diversity parameters were inferior by a factor 2 in *V. destructor* compared to *V. jacobsonii* (Tables S4 and S5, Fig. 3). Given the low observed level of heterozygosity (*H*_o_, Table S6), none of the populations sampled were at a Hardy–Weinberg equilibrium. Notably, *H*_o_ was higher by a factor 2 in *V. jacobsonii* than in *V. destructor*, again with the exception of *V. jacobsonii* Samui2 (Table S6, Fig. 3). The *V. jacobsonii* in the North had the high allelic richness typical of the other continental *V. jacobsonii* but associated with heterozygosity values as low as that of the island population (Fig. 3).

**Fig. 4.**
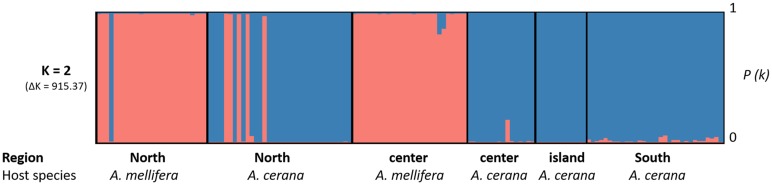
Results of population structure InStruct analysis of *Varroa* spp. mites infesting *A. cerana* and *A. mellifera* in Thailand. The *Y*-axis represents the likelihood for each individual to belong to a genetic cluster. Each cluster is represented by a distinct colour. The *X*-axis shows the different individuals, their location (North, centre, South or island) and host (*Apis mellifera* or *Apis cerana*).

The analysis of population structure using InStruct showed that the most likely number of clusters in our dataset is *K* = 2 (ΔK2 = 915.4). Plotting the individuals belonging to the two genetic clusters revealed a strong host specificity at most locations (Fig. 4). However, untypical host–parasite pairs (*N* = 5 in *A. cerana* and *N* = 1 in *A. mellifera*) were detected in the North of the country (Fig. 4). Since a portion of the individuals that were genotyped were also sequenced, microsatellite data could be associated with mtDNA haplotypes (Table S7). To do so, the nucleotide BLAST tool was used to match the individuals to known *Varroa* species. This analysis revealed that the individuals sampled in *A. mellifera* colonies shared >99.70% identity with *V. destructor* (GenBank accession GQ379056.1, 100% query cover) and that mites sampled in *A. cerana* colonies shared >98.90% identity with *V. jacobsonii* (GQ387678.1, 96–98% query cover, Table S7).

Individuals with a probability of belonging simultaneously to the two clusters above 5% (likely hybrids) were found in *A. cerana* in the North (*N* = 1) and the South (*N* = 1) and in both hosts in the centre (*N* = 3, Fig. 4, Table S8). Lowering the cut-off to 2.5% revealed five additional less likely hybrids in the southern population of *A. cerana*. Visual inspection of the genotype of these individuals confirmed the presence of shared alleles in all these individuals and identified an additional seven putative hybrids. Shared alleles occurred at one or at up to three markers simultaneously in these individuals (Table S8, Tables 1 and 2).

**Table 2.** Allele frequencies per microsatellite locus and ratio of frequencies between *V. destructor* and *V. jacobsonii*

		Species		Ratio
Locus	Allele size	*V. destructor*	*V. jacobsoni*	Higher/lower frequency
VD305	110		5.1	
	113*	1.0	6.8	7.1
	116		20.5	
	119		19.9	
	122	28.8		
	125*	1.0	12.5	13.0
	128		34.1	
	131	61.5		
	134	7.7		
	137		1.1	
VD307	163	24.0		
	165**	75.0	2.2	33.4
	167		0.6	
	169		61.8	
	171		2.2	
	173		12.4	
	175*	1.0	20.2	21.0
	179		0.6	
VD112	133		1.2	
	135	2.1		
	137***	4.3	1.7	2.4
	139**	46.8	6.4	7.3
	141		22.1	
	143		5.2	
	145***	46.8	22.1	2.1
	147		16.9	
	149		3.5	
	151		3.5	
	153		15.7	
	159		1.7	
VJ292	210		100.0	
	232	2.9		
	234	97.1		
VJ294	150		1.6	
	164		9.5	
	166		19.8	
	168		4.0	
	172		12.7	
	174**	98.1	9.5	10.3
	176		1.6	
	178*	1.9	14.3	7.4
	180		5.6	
	182		15.1	
	186		4.0	
	192		2.4	
Vdes-01	400**	41.8	1.2	34.3
	402*	3.1	98.8	32.3
	406	55.1		
Vdes-02	296	71.6		
	308	28.4		
	310		11.6	
	312		57.5	
	314		21.2	
	316		6.2	
	318		1.4	
	330		2.1	
Vdes-04	271***	49.0	10.9	4.5
	273**	51.0	3.8	13.3
	275		6.4	
	277		17.9	
	279		13.5	
	281		7.7	
	283		7.7	
	285		1.3	
	287		8.3	
	289		0.6	
	291		0.6	
	293		3.2	
	299		2.6	
	301		0.6	
	303		10.3	
	305		4.5	

*: alleles rare in *V. destructor* but common in *V. jacobsonii*; **: alleles rare in *V. jacobsonii* but common in V. destructor (ratio of higher/lower frequency >5). ***: alleles common to both species but of uncertain origin (ratio of higher/lower frequency <5).

**Fig. 5.**
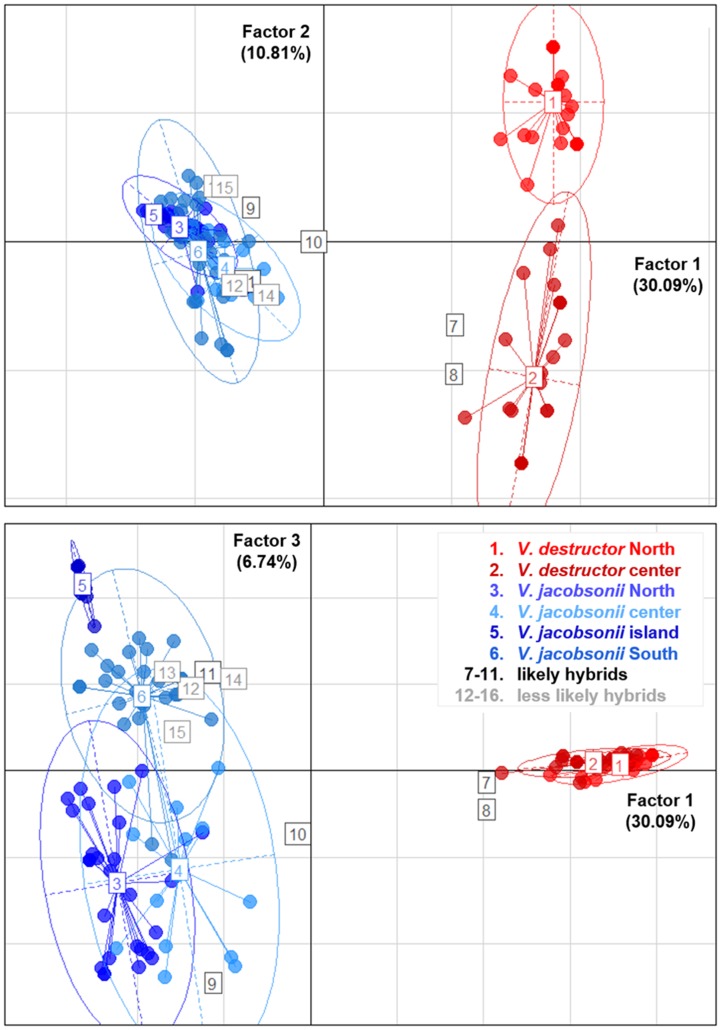
Principal Component Analyses. Genetic clustering based on eight microsatellite markers of mite populations occurring in four regions of Thailand and parasitizing imported *Apis mellifera* (*Varroa destructor*, shades of red) and endemic *Apis cerana* (*Varroa jacobsonii*, shades of blue). The three factors explaining most of the variance are plotted. Percentage of explained variance is indicated on each axis. Putative hybrids identified by InStruct are indicated with numbered squares from 7 to 16. Ellipses represent 95% confidence intervals.

The PCA placed most of the putative hybrids between *V. jacobsonii* and *V. destructor*, along the axis 1, which separated the species, and which explained 30% of the genetic variance (Fig. 5). These individuals did not cluster half way between their suspected parental groups because they only showed heterospecific alleles at one to two loci and were thus closer to the parent they shared most alleles with. Four of them (corresponding to the individuals defined as likely hybrids, Tables 1 and S8) clustered outside the 95% confidence ellipses of their parent groups (Fig. 5). All other suspected hybrids (likely and less likely hybrids) lied within the ellipses. Factor 2 of the PCA explained 11% of the variance and separated the northern from the central *V. destructor* populations. Factor 3 (7% of the variance) did not separate *V. jacobsonii* from the North and the centre of Thailand, but these two populations were separated from the island and the South populations on the third axis (Fig. 5).

**Fig. 6.**
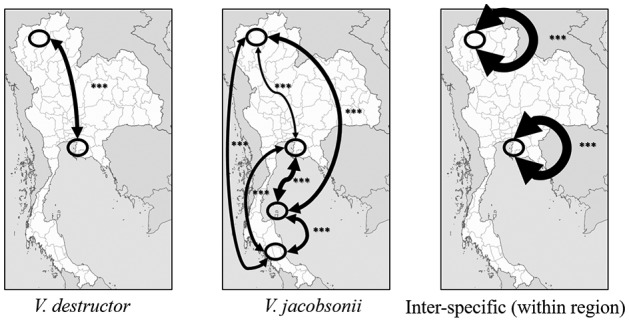
*D*_est_ values between mite populations at different locations in Thailand and host species. Thickness of arrows on the maps is proportional to *D*_est_ value. Intraspecific *D*_est_ values are presented for each host species as well as for the interspecific comparison in the North and the centre. Ellipses designate sampled locations. From North to South: Chiang Mai, Bang Saen, Ko Samui, Phattalung. Statistical differences (1000 bootstrap) of *D*_est_ values between populations are denoted with asterisks (*** *P* < 0.001).

The pairwise comparison of population differentiation estimates (*F*_ST_, *G*_ST_ and *D*_est_) showed strong significant differences between the mites parasitizing the two host species in the northern and central locations (Table S9, Fig. 6). Differences between mites parasitizing *A. mellifera* in the two regions were strong and significant (Table S5). In mites infesting *A. cerana*, the population differentiation estimates were highest when comparing the continental populations (North, centre and South) to the island population (Table S9, Fig. 6).

The AMOVA results indicated that genetic differences among mites infesting colonies of the same location were the least important in *A. cerana* and *A. mellifera* (13.8 and 5.3%, respectively, Table 3). The highest level of genetic structuring was within colonies for mites infesting *A. cerana* (57.4%), and among locations for mites infesting *A. mellifera* (60.9%).

**Table 3.** Results of two independent pairwise distance-based AMOVAs performed with the microsatellite data. Represented is the level of genetic structuring for each host species among locations, colonies and within colonies

Mite species	Host species	Level	d.f.	Sum of square	% Variation	Significance
*V. jacobsonii*		Among locations	3	50.44	28.80	***
	*A. cerana*	Among colonies of the same location	6	16.85	13.79	***
		Within colonies	178	100.83	57.41	***
*V. destructor*		Among locations	1	42.44	60.94	**
	*A. mellifera*	Among colonies of the same location	9	9.46	5.32	n.s.
		Within colonies	89	40.50	33.75	***

Levels of significance were calculated based on 1000 permutations (*** *P* < 0.001; ** *P* < 0.01 and n.s. non-significant).

## Discussion

Our data confirm the *Varroa* spp. haplogroups detected previously (Smith and Hagen, 1996; Warrit *et al*., 2006), but the haplotypes we found differed in 1–4 nucleotides from those described earlier. The reproductive status of the sampled mites further confirmed that these haplotypes were indeed parasites of the host populations they were collected from. Spillbacks of *V. destructor* to *A. cerana* and spillovers of *V. jacobsonii* mites to *A. mellifera* colonies were observed. Genotyping revealed infestation of single host colonies with both *V. destructor* and *V. jacobsonii* as well as with several haplotypes of *V. jacobsonii*, thereby setting the stage for hybridization, which the microsatellites indicated in up to 17 out of 147 mites genotyped.

### Distribution, reproduction and genetic diversity of *Varroa* spp. in Thailand

All haplogroups reported earlier (Warrit *et al*., 2006; Navajas *et al*., 2010) were confirmed. Yet, our choice to sequence a mitochondrial genome region common to several previous studies (Warrit *et al*., 2006; Navajas *et al*., 2010) led to compromises in mite identification. For instance, the V1 and C2 haplotypes and hence the V and C haplogroups of *V. destructor* could not be distinguished from each other using the chosen region. Since the V1 haplotype was reported earlier (Warrit *et al*., 2006), it seems likely that this is the *V. destructor* haplogroup, which we sampled in the North of Thailand. Interestingly, the Japanese *V. destructor* haplogroup was not detected, emphasizing that its presence in Thailand is dubious (Warrit *et al*., 2006). The distribution patterns of the remaining haplogroups described by Warrit *et al*. (2006) were confirmed in broad terms based on reproductive data and genotyping: *V. destructor* K1 was found to infest *A. mellifera*, while *V. jacobsonii* North Thai, Malay and Samui infested *A. cerana* in the North (Chiang Mai), South (Phattalung) and on Samui Island, respectively.

The *V. jacobsonii* haplotypes detected here varied from one to four nucleotides compared to those described over a decade earlier (Warrit *et al*., 2006). The haplotype network indicates that the novel NorthThai3 haplotype of *V. jacobsonii* has not emerged recently because of its intermediate position between the NorthThai2 and Laos1 haplotypes, but has probably not been sampled previously. Despite the collection of a high number of mites in the same region, some previously described haplotypes (Warrit *et al*., 2006) were not confirmed, which could be due to a sampling bias. Indeed, the haplotype network inferred the existence of a few non-sampled haplotypes. Irrespective of their cause, the differences between studies and the high genetic diversity measured suggest that the mite population structure is dynamic in time or space. A more accurate description of the population structure and dynamics of *Varroa* spp. in their original range thus requires even higher sampling efforts.

The number of mites we sampled nevertheless allowed for the detection of unexpected host–parasite associations. Reproduction of the Korea haplotype of *V. destructor* was repeatedly observed for the first time on *A. cerana* drone brood outside of its natural range, thereby demonstrating a lower host specificity than previously suspected (see Navajas *et al*., 2010). Possible differences in drone brood entombing (Rath, 1999) and/or susceptibility of host worker brood (Page *et al*., 2016) between populations may explain why this particular lineage has not invaded all *A. cerana* populations sympatric with infested *A. mellifera*. Despite the ubiquity of imported *A. mellifera* in Asia, none of the studies investigating population genetics in *Varroa* spp. reported the invasive Korean lineage of *V. destructor* (K1) infesting *A. cerana* outside its original distribution range (Anderson and Trueman, 2000; Fuchs *et al*., 2000; Solignac *et al*., 2005; Navajas *et al*., 2010; Beaurepaire *et al*., 2015). The number of surveys remains small and spillbacks of the *V. destructor* Korea haplotype into non-native host populations of *A. cerana* could have gone undetected. Yet, the spillback of the virulent *V. destructor* lineage or its hybridization with endemic mites could have dramatic consequences for populations of *A. cerana* (Depotter *et al*., 2016). Moreover, its propensity to vector a large diversity of viruses represents a threat to honey bees and other pollinators (Fürst *et al*., 2014; Wilfert *et al*., 2016).

Using mtDNA sequencing, we also detected the occurrence of a single *V. jacobsonii* mite spillover from *A. cerana* to *A. mellifera*. The observed frequency of 1% (once in 117 cases), suggests that opportunities for host shifts by this mite species (e.g. in Papua New Guinea, Roberts *et al*., 2015) are not extremely rare. Whether this *V. jacobsonii* mite reproduced on its *A. mellifera* host could not be established. Nevertheless, this finding is alarming and highlights the risks of host shifts by mite lineages of further haplogroups.

Drifting of mites between host species, as well as the natural sympatry of several haplotypes of mites, can lead to infestations of single host colonies and even brood cells by mites of multiple haplotypes and species, thereby setting the stage for hybridization. Indeed, such cases were detected in five *A. cerana* colonies and in one *A. mellifera* colony. In addition, the relatively high frequency of multiply infested drone cells in these populations (up to 13% of infested cells, Wang *et al*., in prep.) supports the idea that opportunities for hybridization can indeed be frequent.

### Putative introgression between mite species

The occurrence of several species and haplotypes in single colonies leads to the possibility of foundress mites of different taxa entering the same host brood cell to reproduce (Beaurepaire *et al*., 2017*a*). The cohabitation of their sexually mature offspring sets the stage for hybridization. Indeed, our analysis with Instruct revealed that five likely hybrids could not unambiguously be assigned to a single genetic cluster. In complement, visual inspection of the genotypes of these individuals revealed that they carried alleles usually found on mites infesting the other host species at up to three markers (Table S8). On the PCA, some of the likely hybrids were also found outside of the 95% confidence ellipses of their group. However, given that the Adegenet PCA function incorporates missing data as averaged alleles, the other individuals (likely and less likely hybrids) carrying heterospecific alleles could not be clearly distinguished from the rest using this method. Although the presence of an identical allele in the mite species may be due to size homoplasy, this event alone does not seem sufficient to explain the patterns we observed, with the presence of shared alleles in six loci out of eight (Table S8). The large size difference with a putative parent allele strengthens our argument. For instance, we found the allele ‘175’ at locus VD307 in an individual with a dominant *V. destructor* genotype and 99.88% identity to the K1 COI haplotype (Tables S7 and S8). The closest allele found in the gene pool of *V. destructor* is 165 (75% prevalence). With a repeat motif of this microsatellite of 2 bp, at least five additions/deletions would be necessary to generate homoplasy, which seem highly unlikely.

The introgression of alleles of *V. destructor* in the gene pool of *V. jacobsonii* from the centre and the South of Thailand at some but not all loci suggests the presence of second or third generation hybrids and indicates that the two species are capable of interbreeding and of producing fertile offspring. Introgression suggests that reproductive barriers between these species are absent and questions the segregation of *V. destructor* and *V. jacobsonii* into two species. Differences in behaviour, morphology and virulence promoted the investigation of genetic divergence within the genus *Varroa* (Anderson and Fuchs, 1998; de Guzman *et al*., 1998; Anderson, 2000). The percentage of divergence measured resulted in the definition of *V. destructor* as a new species (Anderson and Trueman, 2000). Yet, whether the typical biological basis for species definition is fulfilled (i.e. the absence of interbreeding and production of fertile offspring, Mayr, 1942) has never been tested. Therefore, the potential of hybridization between these two mite species needs to be investigated experimentally to provide direct evidence of what our data suggest. In case it is confirmed, the relative scarcity of hybrids found to date requires an explanation. Post-zygotic isolation mechanisms, for example, have been suggested to limit the occurrence of hybrids between the Korea and Japan haplotypes of *V. destructor* (Solignac *et al*., 2005).

### Population structure and reproductive system

The difference between expected and observed heterozygosity (Table S6) indicates that the sampled *Varroa* populations were not in Hardy–Weinberg equilibrium, which is in line with the mites' inbred reproductive system (Rosenkranz *et al*., 2010). This mating system also explains the low levels of genetic diversity, which were further exacerbated by a genetic bottleneck due to host shift (for the invasive *V. destructor*, Solignac *et al*., 2005) and to isolation on an island (*V. jacobsonii* Samui2; Oldroyd and Wongsiri, 2006). As a result of these bottlenecks, the number of alleles and allelic richness of these populations was inferior to that of the mites in other *A. cerana* populations. A notable inconsistency was observed for the northern (Chiang Mai) population, which showed the high range in allelic richness typical for *V. jacobsonii* (except Samui2), but a lower range of heterozygosity (similar to Samui2; Fig. 5). This suggests a higher inbreeding rate in this population, but without loss of allelic richness, of which the causes remain unknown. Overall, the range of genetic diversity of the *V. jacobsonii* populations in Thailand was similar to that found in other populations of this mite taxon (Roberts *et al*., 2015).

Our investigations of the population structure with microsatellite markers show that the gene flow between *V. destructor* and *V. jacobsonii* was overall low (Table S5) but may be mediated by occasional hybridization. The comparison of the different indexes of population differentiation revealed contrasting patterns in the mites infesting the two host species. The two indexes *F*_ST_ and *G*_ST_ were generally higher in *A. mellifera* than *D*_est_. This trend was reversed for the mite populations infesting *A. cerana*. These discrepancies can be explained by the differences in the number of alleles and heterozygosity levels within these two groups (Meirmans and Hedrick, 2011), with *V. destructor* subpopulations being less diverse than those of *V. jacobsonii*.

The pattern of genetic structure among the *A. cerana* mite populations mostly correlated with geographic distance and isolation (Table S5, Figs 4–6). In the native host, mite subpopulations from the continent may have exchanged alleles frequently, probably as a consequence of *A. cerana* colonies migrating seasonally (Oldroyd and Wongsiri, 2006). Notably, lower levels of genetic differentiation were detected between mites from the North and the centre compared to the mites from the South (Figs 5 and 6), probably reflecting mite adaptation to the local host haplotypes (Rueppell *et al*., 2011). However, we found evidence of nuclear gene flow across the Kra Isthmus, which physically separates *A. cerana* Mainland and Sundaland subpopulations. These results support the hypothesis that host–parasite associations in the *Apis*–*Varroa* system are not only due to local coevolution, but can be influenced by biogeographic history and population migration (Rueppell *et al*., 2011). The genetic distinctiveness of the Samui island mite population mirrors its host's geographical isolation (see Rueppell *et al*., 2011). Gene flow between the continental and the Samui populations was likely interrupted 18 000 to 10 000 years ago as the sea level rose (Oldroyd and Wongsiri, 2006). Using the substitution rates proposed by Solignac *et al*. (2005), this timespan corresponds to a range of 3–14 substitutions on the COI gene when comparing the island with the other *V. jacobsonii* haplotypes, fitting with our mtDNA results (Fig. 2).

In accordance with the genetic differentiation estimates, the two AMOVAs revealed different patterns of genetic structuring in *A. mellifera* mites and in mites of the native host. The gene flow of the new host is likely a consequence of human transportation of colonies, as feral colonies of *A. mellifera* do not occur in Asia (Oldroyd and Wongsiri, 2006). Although trade and the associated translocation of hosts along the country's North-South axis (Chantawannakul, 2018) could be expected to level out population structuring in the parasite, we found high population differentiation levels in mites infesting *A. mellifera* (Figs 5 and 6). These may be due to mite introductions of different origins and genotypes and/or due to local adaptation.

Additionally, we detected a low genetic structuration among colonies of the same location in *V. jacobsonii and V. destructor*, most likely reflecting that mites readily disperse among colonies (Dynes *et al*., 2016; Beaurepaire *et al*., 2017*b*). The analysis of genetic structure at the lowest scale (within colony) revealed that the genetic diversity between *V. jacobsonii* mite infesting the same colonies was considerable. Indeed, the *V. jacobsonii* genotypes in *A. cerana* colonies were sampled only once (Table S8). In contrast, the moderate genetic variance at this level in *V. destructor* reflects the lack of diversity of the Korea haplotype outside its natural distribution (Solignac *et al*., 2005). Altogether, given the peculiar patterns of *Varroa* population structure, varying in space, time and according to its host species, a broader sampling scheme will be necessary to seize the extent of this parasite's complex biogeography in Asia.

## Conclusion

Several of the phenomena known to promote host shifts have been observed in our screening of natural infestations of *A. cerana* and *A. mellifera* by *V. jacobsonii* and *V. destructor*. Genetic diversity of *V. jacobsonii* was higher compared to *V. destructor*. Spillbacks of invasive *V. destructor* mites from *A. mellifera* into *A. cerana* and spillovers of endemic *V. jacobsonii* mites from *A. cerana* to introduced *A. mellifera* were observed. These events resulted in infestations of single colonies with both mite species and microsatellite marker based evidence suggested hybridization between *V. destructor* and *V. jacobsonii*. The relatively high frequency of these phenomena indicate risks of further host shifts, which could threaten honey bee populations of Asia and beyond.

## Supplementary material

For supplementary material accompanying this paper visit https://doi.org/10.1017/S003118201900091X. click here to view supplementary material

## References

[ref1] Altschul SF, Gish W, Miller W, Myers EW and Lipman DJ (1990) Basic local alignment search tool. Journal of Molecular Biology 215, 403–410. 2231712 10.1016/S0022-2836(05)80360-2

[ref2] Anderson DL (2000) Variation in the parasitic bee mite *Varroa jacobsoni* Oud. Apidologie 31, 281–292.

[ref3] Anderson DL and Fuchs S (1998) Two genetically distinct populations of *Varroa jacobsoni* with contrasting reproductive abilities on *Apis mellifera*. Journal of Apicultural Research 37, 69–78.

[ref4] Anderson DL and Trueman JWH (2000) *Varroa jacobsoni* (Acari: Varroidae) is more than one species. Experimental and Applied Acarology 24, 165–189. 11108385 10.1023/a:1006456720416

[ref5] Beaurepaire AL, Truong TA, Fajardo AC, Dinh TQ, Cervancia C and Moritz RFA (2015) Host specificity in the honeybee parasitic mite, *Varroa* spp. in *Apis mellifera* and *Apis cerana*. PLoS ONE 10, e0135103. 26248192 10.1371/journal.pone.0135103 PMC4527838

[ref6] Beaurepaire AL, Krieger KJ and Moritz RFA (2017 *a*) Seasonal cycle of inbreeding and recombination of the parasitic mite *Varroa destructor* in honeybee colonies and its implications for the selection of acaricide resistance. Infection Genetics and Evolution 50, 49–54. 10.1016/j.meegid.2017.02.011 28216419

[ref7] Beaurepaire AL, Ellis JD, Krieger KJ and Moritz RFA (2017b) Association of *Varroa destructor* females in multiply infested cells of the honeybee *Apis mellifera*. Insect science 26, 128–134. 28834265 10.1111/1744-7917.12529

[ref8] Chantawannakul P (2018) Bee diversity and current status of beekeeping in Thailand In Chantawannakul P, Williams G and Neumann P (eds), Asian Beekeeping in the 21^st^ Century. Singapore: Springer, 325 pp. doi: 10.1007/978-981-10-8222-1.

[ref9] Criscione DC, Poulin R and Blouin MS (2005) Molecular ecology of parasites: elucidating ecological and microevolutionary processes. Molecular Ecology 14, 2247–2225. 15969711 10.1111/j.1365-294X.2005.02587.x

[ref10] de Guzman L, Rinderer TE, Stelzer JA and Anderson D (1998) Congruence of RAPD and mitochondrial DNA markers in assessing *Varroa jacobsoni* genotypes. Journal of Apicultural Research 37, 49–51.

[ref11] de Meeûs T, McCoy K, Prugnolle F, Chevillon C, Durand P, Hurtrez-Boussès S and Renaud F (2007) Population genetics and molecular epidemiology or how to ‘débusquer la bête’. Infection, Genetics and Evolution 7, 308–332. 10.1016/j.meegid.2006.07.003 16949350

[ref12] Depotter JRL, Seidl MF, Wood TA and Thomma BPHJ (2016) Interspecific hybridization impacts host range and pathogenicity of filamentous microbes. Current Opinion in Microbiology 32, 7–13. 27116367 10.1016/j.mib.2016.04.005

[ref13] Dietemann V, Pflugfelder J, Anderson D, Charrière J-D, Chejanovsky N, Dainat B, de Miranda J, Delaplane K, Dillier F-X, Fuch S, Gallmann P, Gauthier L, Imdorf A, Koeniger N, Kralj J, Meikle W, Pettis J, Rosenkranz P, Sammataro D, Smith D, Yañez O and Neumann P (2012) *Varroa destructor*: research avenues towards sustainable control. Journal of Apicultural Research 51, 125–132.

[ref14] Dietemann V, Nazzi F, Martin SJ, Anderson D, Locke B, Delaplane KS, Wauquiez Q, Tannahill C, Frey E, Ziegelmann B, Rosenkranz P and Ellis JD (2013) Standard methods for varroa research. In Dietemann V, Ellis JD, Neumann P (eds.). The COLOSS *BEEBOOK*, Volume II: standard methods for *Apis mellifera* pest and pathogen research Journal of Apicultural Research 52. doi: 10.3896/IBRA.1.52.1.09.

[ref15] Dynes TL, De Roode JC, Lyons JI, Berry JA, Delaplane KS and Brosi BJ (2016) Fine scale population genetic structure of *Varroa destructor*, an ectoparasitic mite of the honey bee (*Apis mellifera*). Apidologie 48, 93–101. 10.1007/s13592-016-0453-7 PMC5089174 27812229

[ref16] Evanno G, Regnault S and Goudet J (2005) Detecting the number of clusters of individuals using the software structure: a simulation study. Molecular Ecology 14, 2611–2620. 15969739 10.1111/j.1365-294X.2005.02553.x

[ref17] Evans JD (2000) Microsatellite loci in the honey bee parasitic mite *Varroa jacobsoni*. Molecular Ecology 9, 1436–1438. 10972785 10.1046/j.1365-294x.2000.00998-3.x

[ref18] Evans JD, Schwarz RS, Chen YP, Budge G, Cornman RS, de la Rúa P, de Miranda JR, Foret S, Foster L, Gauthier L, Genersch E, Gisder S, Jarosch A, Kucharski R, Lopez D, Lun CM, Moritz RFA, Maleszka R, Muñoz I and Pinto MA (2013) Standard methodologies for molecular research in *Apis mellifera*. In Dietemann V, Ellis JD and Neumann P (eds.) The COLOSS *BEEBOOK*, Volume I: standard methods for *Apis mellifera* research Journal of Apicultural Research 52. doi: 10.3896/IBRA.1.52.4.11.

[ref19] Excoffier L, Laval G and Schneider S (2005) Arlequin (version 3.0): an integrated software package for population genetics data analysis. Evolutionary Bioinformatics 1, 47–50. PMC2658868 19325852

[ref20] Fuchs S, Tu Long L and Anderson D (2000) A scientific note on the genetic distinctness of *Varroa* mites on *Apis mellifera* L. and on *Apis cerana* Fabr. in North Vietnam. Apidologie 31, 459–460.

[ref21] Fürst MA, McMahon DP, Osborne JL, Paxton RJ and Brown MJF (2014) Disease associations between honeybees and bumblebees as a threat to wild pollinators. Nature 506, 364–366. 24553241 10.1038/nature12977 PMC3985068

[ref22] Gao H, Williamson S and Busta-mante CD (2007) An MCMC approach for joint inference of population structure and inbreeding rates from multi-locus genotype data. Genetics 176, 1635–1651. 17483417 10.1534/genetics.107.072371 PMC1931536

[ref23] Genersch E, Von der Ohe V, Kaatz H, Schroeder A, Otten C, Büchler R, Berg S, Ritter W, Mühlen W, Gisder S, Meixner M, Liebig G and Rosenkranz P (2010) The German bee monitoring project: a long term study to understand periodically high winter losses of honey bee colonies. Apidologie 41, 332–352.

[ref24] Goudet J (1995) FSTAT (version 1.2): a computer program to calculate F-statistics. Journal of Heredity 86, 485–486.

[ref25] Guzmán-Novoa E, Eccles L, Calvete Y, Mcgowan J, Kelly PG and Correa-Benìtez A (2010) *Varroa destructor* is the main culprit for the death and reduced populations of overwintered honey bee (*Apis mellifera*) colonies in Ontario, Canada. Apidologie 41, 443–450.

[ref26] Harrison RG (1989) Animal mitochondrial DNA as a genetic marker in population and evolutionary biology. Trends in Ecology and Evolution 4, 6–11. 21227301 10.1016/0169-5347(89)90006-2

[ref27] Jaffé R, Dietemann V, Allsopp MH, Costa C, Crewe RM, Dall'Olio R, de la Rúa P, El-Niweiri MAA, Fries I, Kezic N, Meusel M, Paxton RJ, Shaibi T, Stolle E and Moritz RFA (2009) Estimating the density of honeybee colonies across their natural range to fill the gap in pollinator decline censuses. Conservation Biology 24, 583–593. 19775273 10.1111/j.1523-1739.2009.01331.x

[ref28] Jakobsson M and Rosenberg NA (2007) CLUMPP: a cluster matching and permutation program for dealing with label switching and multimodality in analysis of population structure. Bioinformatics (Oxford, England) 23, 1801–1906. 10.1093/bioinformatics/btm233 17485429

[ref29] Jombart T (2008) Adegenet: a R package for the multivariate analysis of genetic markers. Bioinformatics (Oxford, England) 24, 1403–1405. 10.1093/bioinformatics/btn129 18397895

[ref30] Jost L (2008) GST and its relatives do not measure differentiation. Molecular Ecology 17, 4015–4026. 19238703 10.1111/j.1365-294x.2008.03887.x

[ref31] Kleijn D, Winfree R, Bartomeus I, Carvalheiro LG, Henry M, Isaacs R, Klein A-M, Kremen C, Gonigle LKM, Rader R, Ricketts TH, Williams NM, Adamson NL, Ascher JS, Báldi A, Batáry P, Benjamin F, Biesmeijer JC, Blitzer EJ, Bommarco R, Brand MR, Bretagnolle V, Button L, Cariveau DP, Chifflet R, Colville JF, Danforth BN, Elle E, Garratt MPD, Herzog F, Holzschuh A, Howlett BG, Jauker F, Jha S, Knop E, Krewenka KM, Le Féon V, Mandelik Y, May EA, Park MG, Pisanty G, Reemer M, Riedinger V, Rollin O, Rundlöf M, Sardiñas HS, Scheper J, Sciligo AR, Smith HG, Steffan-Dewenter I, Thorp R, Tscharntke T, Verhulst J, Viana BF, Vaissière BE, Veldtman R, Westphal C and Potts SG (2015) Delivery of crop pollination services is an insufficient argument for wild pollinator conservation. Nature Communications 6, 7414. 10.1038/ncomms8414 PMC4490361 26079893

[ref32] Klein A-M, Vaissière BE, Cane JH, Steffan-Dewenter I, Cunningham SA, Kremen C and Tscharntke T (2007) Importance of pollinators in changing landscapes for world crops. Proceedings of the Royal Society B 274, 303–313. 17164193 10.1098/rspb.2006.3721 PMC1702377

[ref33] Kolar CS and Lodge DM (2001) Progress in invasion biology: predicting invaders. Trends in Ecology and Evolution 16, 199–204. 11245943 10.1016/s0169-5347(01)02101-2

[ref34] Kumschick S, Bacher S, Evans T, Markova Z, Pergl J, Pysek P, Vaes-Petignat S, van der Veer G, Vila M and Nentwig W (2015) Comparing impacts of alien plants and animals in Europe using a standard scoring system. Journal of Applied Ecology 52, 552–561.

[ref35] Le Conte Y, de Vaublanca G, Crausera D, Jeanne F, Roussellec J-C and Bécarda J-M (2007) Honey bee colonies that have survived *Varroa destructor*. Apidologie 38, 566–572.

[ref36] Le Conte Y, Ellis M and Ritter W (2010) Varroa mites and honey bee health: can Varroa explain part of the colony losses? Apidologie 41, 353–363.

[ref37] Longdon B, Brockhurst MA, Russell CA, Welch JJ and Jiggins FM (2014) The evolution and genetics of virus host shifts. PLoS Pathogen 10, e1004395. 25375777 10.1371/journal.ppat.1004395 PMC4223060

[ref38] Mayr E (1942) Systematics and the Origin of Species. New York: Columbia University Press.

[ref39] Meirmans PG and Hedrick PW (2011) Assessing population structure: FST and related measures. Molecular Ecology Resources 11, 5–18. 21429096 10.1111/j.1755-0998.2010.02927.x

[ref40] Navajas M, Anderson DL, de Guzman LI, Huang Z-Y, Clément J, Zhou T and Le Conte Y (2010) New Asian types of *Varroa destructor*: a potential new threat for world apiculture. Apidologie 41, 181–193.

[ref41] Nei M (1973) Analysis of gene diversity in subdivided populations. Proceedings of the National Academy of Sciences of the USA 70, 3321–3323. 4519626 10.1073/pnas.70.12.3321 PMC427228

[ref42] Nguyen BK, Ribière M, vanEngelsdorp D, Snoeck C, Saegerman C, Kalkstein AL, Schurr F, Brostaux Y, Faucon J-P and Haubruge E (2011) Effects of honey bee virus prevalence, *Varroa destructor* load and queen condition on honey bee colony survival over the winter in Belgium. Journal of Apicultural Research 50, 195–202.

[ref43] Oldroyd BP and Wongsiri S (2006) Asian Honey Bees. Biology, Conservation, and Human Interactions. Cambridge, MA: Harvard University Press.

[ref44] Page P, Lin ZG, Buawangpong N, Zheng HQ, Hu FL, Neumann P, Chantawannakul P and Dietemann V (2016) Social apoptosis in honey bee superorganisms. Scientific Reports 6, 27210. 27264643 10.1038/srep27210 PMC4893659

[ref45] Peakall R and Smouse PE (2012) Genalex 6.5: genetic analyses in Excel. Population genetic software for teaching and research – an update. Bioinformatics (Oxford, England) 28, 2537–2539. 10.1093/bioinformatics/bts460 PMC3463245 22820204

[ref46] Pimentel D, Zuniga R and Morrison D (2005) Update on the environmental and economic costs associated with alien-invasive species in the United States. Ecological Economics 52, 273–288.

[ref47] Pritchard JK, Stephens M and Donnelly P (2000) Inference of population structure using multilocus genotype data. Genetics 155, 945–959. 10835412 10.1093/genetics/155.2.945 PMC1461096

[ref48] R Core Team (2018) R: a language and environment for statistical computing. R Foundation for Statistical Computing, Vienna.

[ref49] Rath W (1999) Co-adaptation of *Apis cerana* Fabr. and *Varroa jacobsoni* Oud. Apidologie 30, 97–110.

[ref50] Roberts JMK, Anderson DL and Tay WT (2015) Multiple host shifts by the emerging honeybee parasite, *Varroa jacobsoni*. Molecular Ecology 24, 2379–2391. 25846956 10.1111/mec.13185

[ref51] Rosenberg NA (2004) Distruct: a program for the graphical display of population structure. Molecular Ecology 4, 137–138.

[ref52] Rosenkranz P, Aumeier P and Ziegelmann B (2010) Biology and control of *Varroa destructor*. Journal of Invertebrate Pathology 103, S96–S119. 19909970 10.1016/j.jip.2009.07.016

[ref53] Rueppell O, Hayes AM, Warrit N and Smith DR (2011) Population structure of *Apis cerana* in Thailand reflects biogeography and current gene flow rather than *Varroa* mite association. Insectes Sociaux 58, 445–452.

[ref54] Smith DR and Hagen RH (1996) The biogeography of *Apis cerana* as revealed by mitochondrial DNA sequence data. Journal of the Kansas Entomological Society 69, 294–310.

[ref55] Smith KM, Loh EH, Rostal MK, Zambrana-Torrelio CM, Mendiola L and Daszak P (2013) Pathogens, pests, and economics: drivers of honey bee colony declines and losses. EcoHealth 10, 434–445. 24496582 10.1007/s10393-013-0870-2

[ref56] Solignac M, Cornuet JM, Vautrin D, Le Conte Y, Anderson D, Evans J, Cros-Arteil S and Navajas M (2005) The invasive Korea and Japan types of *Varroa destructor*, ectoparasitic mites of the Western honeybee (*Apis mellifera*), are two partly isolated clones. Proceedings of the Royal Society Biological Sciences 272, 411–419. 15734696 10.1098/rspb.2004.2853 PMC1634981

[ref58] Thompson JN (1994) The Coevolutionary Process. Chicago, USA: University of Chicago Press.

[ref59] Walsh PS, Metzger DA and Higuchi R (1991) Chelex 100 as a medium for simple extraction of DNA for PCR-based typing from forensic material. Biotechnique 10, 506–513. 1867860

[ref60] Wang S, Lin Z, Dietemann V, Neumann P, Wu YQ, Hu FL and Zheng HQ (2018) Ectoparasitic mites *Varroa underwoodi* (Acarina: Varroidae) in Eastern, but not in Western honeybees. Journal of Economic Entomology 112, 25–32. 10.1093/jee/toy288 30277506

[ref61] Warrit N, Smith DR and Lekprayoon C (2006) Genetic subpopulations of *Varroa* mites and their *Apis cerana* hosts in Thailand. Apidologie 37, 19–30.

[ref62] Wells K and Clark NJ (2019) Host specificity in variable environments. Trends in Parasitology 35, 452–465. 31047808 10.1016/j.pt.2019.04.001

[ref63] Whitlock MC (2011) G’_ST_ and D do no_t_ replace F_ST_. Molecular Ecology 20, 1083–1091. 21375616 10.1111/j.1365-294X.2010.04996.x

[ref64] Wilfert L, Long G, Leggett HC, Schmid-Hempel P, Butlin R, Martin SJM and Boots M (2016) Deformed wing virus is a recent global epidemic in honeybees driven by Varroa mites. Science 351, 594–597. 26912700 10.1126/science.aac9976

[ref66] Woolhouse ME, Haydon DT and Antia R (2005) Emerging pathogens: the epidemiology and evolution of species jumps. Trends in Ecology and Evolution 20, 238–244. 16701375 10.1016/j.tree.2005.02.009 PMC7119200

